# Εffect of cleansers on the composition and mechanical properties of orthodontic aligners in vitro

**DOI:** 10.1186/s40510-022-00449-w

**Published:** 2022-12-15

**Authors:** Anna Iliadi, Vera Enzler, Georgios Polychronis, Timo Peltomaki, Spiros Zinelis, Theodore Eliades

**Affiliations:** 1 Department of Biomaterials, School of Dentistry, National and Kapodistrian University of Athens, Athens, Greece; 2 Clinic of Orthodontics and Pediatric Dentistry, Center of Dental Medicine, University of Zurich, Plattenstrasse 11, 8032 Zurich, Switzerland; 3 Department of Oral and Maxillofacial Diseases, Tampere University Hospital, Tampere, Finland Faculty of Medicine and Health Technology, Tampere University, Tampere, Finland; 4 Department of Oral and Maxillofacial Diseases, Kuopio University Hospital, Kuopio, Finland; 5 Faculty of Health Sciences, Institute of Dentistry, University of Eastern Finland, Kuopio, Finland

**Keywords:** Cleansers, Aligners, Mechanical properties, Chemical alterations

## Abstract

**Background:**

The aim of the study was to investigate the effect of three aligner cleaners on the composition and mechanical properties of two types of orthodontic aligners.

**Materials and methods:**

The cleaners tested were two alkaline peroxide solutions (Retainer Brite—RB; Retainer Cleaner—RC) and one peroxide-free (Steraligner—ST) and the aligners Clear Aligner (C, polyester) and Invisalign (I, polyester–urethane). The aligners were immersed in the cleaner solutions as instructed every day (15 min for RB, RC; 5 min for ST) for a two-week period. The acidity of the solutions was tested with a pH meter. The changes in the chemical composition of the aligners were studied by attenuated total-reflection Fourier transform infrared spectrometry (ATR-FTIR), while Instrumented Indentation Testing (IIT) was used for assessment of changes in Martens Hardness (HM), modulus (E_IT_), elastic index (n_IT_) and relaxation (R_IT_).

**Results:**

RB and RC were weakly acidic (pH = 6.3), whereas ST was mildly acidic (pH = 4.8). The ATR-FTIR analysis demonstrated evidence of acidic hydrolysis of C in ST and I in RB. The IIT-derived properties of I were not affected by the cleaners. However, for C a significant change was found in HM (all cleaners), n_IT_ (all cleaners) and R_IT_ (RB, ST). Although the chemical changes support a hydrolytic material deterioration, the results of mechanical properties may interfere with the material residual stresses during fabrication.

**Conclusions:**

Caution should be exerted in the selection of aligner cleaners. The mild acidic cleanser was more aggressive to the polyester, whereas an alkaline peroxide to the polyester–urethane aligner.

## Introduction

Aligner system technology provides an orthodontic treatment modality for patients regarding aesthetics highly [1, 2]. The sequential positioners are usually fabricated out of poly(ester–urethane) (PU) or polyethylene terephthalate glycol (PET-G) thermoplastic materials [3] which are translucent and difficult to detect with naked eye. Every aligner becomes deformed upon placement exerting light forces to the teeth. Each removable appliance remains intraorally for two weeks usually, until being replaced by the following new one, inducing tooth movement in an incremental fashion. During this short period, the stability of their properties is of major importance for clinical effectiveness [4].

A major issue with these devices is that the stagnation of salivary flow make their internal surfaces prone to plaque accumulation [5, 6] and staining [7–9]. Calculus formation, although not very common, cannot be excluded, as well. Thus, it becomes imperative for the patients to retain oral hygiene [10] at an appropriate level and remove any debris left in the aligner surfaces. For that purpose, chemical cleaners have been developed requiring no patient dexterity unlike to the toothbrush/toothpaste or soap combination alternatives. These are mild sanitization solutions of various acidity containing sodium bicarbonate, acids, sulfates, chelators and a variety of salts. The cleaners are capable of efficiently removing bacteria biofilms, restoring the original translucency of the appliances and offering a pleasant odor when immersed daily for a few minutes [5, 6, 11–13]. However, the reactivity of the cleaners has raised questions on possible chemical modifications of the aligners and consequently on their mechanical properties, which may adversely affect the treatment outcome. In the relevant literature, the information available for such side effects regarding this interaction is limited and involves mainly thermoplastic retainers [11–15] used to stabilize the orthodontic treatment outcome. In particular, changes were observed in the flexural modulus of chemically cleaned retainers made of copolyester [12], whereas those of polypropylene/ethylene copolymer [13] or polyurethane [14] did not present significant deviations. For aligners, the effect of cleaners on the time-dependent mechanical properties of the devices, which are crucial for the stress-transfer characteristics of the light continuous forces to the teeth, has not been addressed so far.

The aim of the present study was to evaluate the changes in the mechanical properties and surface chemistry of aligners treated with cleaning solutions of different composition. The null hypothesis was that the cleansers have a negligible effect on the properties tested.

## Materials and methods

### Materials

The aligners and the cleaning agents tested are presented in Table 1. Forty unused upper aligners of Clear Aligners (C) and Invisalign (I) aligners were obtained from an orthodontic practice and classified into four groups of ten specimens each per material. The cleansing solutions of RB ad RC were prepared by dissolving each tablet in 150 ml of tap water, whereas for ST 15 ml of the liquid was mixed with 135ml of tap water.

**Table 1:** The aligner materials and the cleaning agents used in the study

Product/code	Composition*	Manufacturer
*Aligners*		
CA Clear Aligner/C	Polyethylene terephthalate glycol	Scheu-Dental GmbH, Iserlohn, Germany
Invisalign/I	Polyester–urethane	Align Technology, San Jose, CA, USA
*Cleaning agents*		
Retainer Brite/RB	Potassium peroxymonosulfate, Sodium perborate monohydrate, Sodium bicarbonate, Sodium sulfate, Sodium carbonate, Pentasodium triphosphate, Corn syrup solids, Sodium lauryl sulfoacetate, PEG-180, Flavor, Magnesium stearate, Tetrasodium EDTA, Citric acid, FD&C Blue #1, FD&C Blue #2	Dentsply Sirona, Sarasota, FL, USA
Retainer Cleaner/RC	Potassium peroxymonosulfate, Sodium percarbonate, PEG-150, Peppermint oil, Indigo, Sodium benzonate, Sodium bicarbonate, Tetrasodium EDTA, Sodium lauryl sulfate	Fancymay, Greenland, (Amazon Associate)
Steraligner/ST	Surfactant, Polyrsorbate 20, Sodium pyrophosphate, Tetrapotassium salt (undefined), Essential oil complex, Sodium gluconate, 2-propanol, Disodium EDTA, Sodium benzonate, Sodium bicarbonate, FD&C Blue #1	TJA Health LLC, Joliet, IL, USA

^*^According to the manufacturers’ information

Aligners designated for Retain Brite (RB) and Retain Cleaner (RC) treatment groups were immersed in individual caps with the cleaning agents for 15 min, whereas a 5-min immersion period was used for the Steraligner (ST) group, according to the manufacturer’s instructions. After each cleansing cycle, the aligners were rinsed thoroughly with tap water and then stored in dry conditions. This procedure was repeated 14 times, once per day for a two-week period, corresponding to a daily cleaning during the instructed in-service function of each appliance. Aligners non-immersed in the cleaning solutions were used as control (CO).

### pH measurements

The pH of 150 ml freshly made cleaning solutions was measured by a calibrated pH meter (P 903, Consort NV, Turnhout, Belgium) employing a standard liquid probe. Measurements were performed two minutes after mixing in triplicate and the values were averaged.

### Mechanical properties (IIT)

Ten upper first molars from different appliances of each testing group (RB, RC, ST, CO) per aligner type (C, I) were sectioned. The specimens were embedded in self-curing acrylic resin (Verso Cit-2, Struers, Ballerup, Denmark), with their occlusal surfaces parallel to the horizontal plane. The samples were ground up to 4000 grit-size SiC papers under water cooling, and polished with a water-based diamond suspension (Nap R1 DiaPro, Struers) in a grinding/polishing machine (Dap-V, Struers). Then, the specimens were subjected to Instrumented Indentation Testing (IIT), employing a universal hardness testing machine (ZHU0.2/Z2.5, Zwick Roell, Ulm, Germany) with a Vickers indenter for determination of the following mechanical properties: the Martens Hardness (HM), indentation modulus (E_IT_), elastic index (n_IT_) which is indicative for the brittleness of the material, and the indentation relaxation (R_IT_). Two different loading regimes were applied. The HM, E_IT_ and n_IT_ were acquired from force–indentation depth curves applying a maximum load of 2.9 N for 2 s contact time. The R_IT_ (monitoring the load level, while maintaining a constant contact area between the indenter and the material) was measured employing a tetragonal force pulse where a constant indentation depth was applied for 60 s and the R_IT_ was measured by recording the force decrease between the start and the end of the constant indentation depth period. All mechanical properties were measured according to the equations provided by the international standard ISO14577-1, 2002 [16].

### Surface chemical composition (ATR-FTIR)

Another series of specimens was prepared by sectioning as above. Intact occlusal specimen surfaces were analyzed by Attenuated Total Reflectance Fourier Transform Infrared Spectrometry (ATR-FTIR), employing a spectrometer (Spectrum GX, PerkinElmer, Buckinghamshire, Bacon, UK) equipped with an ATR accessory (Golden Gate, Specac, Orpington, Kent, UK) with a diamond type III crystal (2 × 2 mm) and a sapphire anvil. Spectra were acquired after under the following conditions: 4000–650 cm^−1^ wavenumber range, 4 cm^−1^ resolution, 20 scans co-addition, 2 μm depth of analysis at 1000 cm^−1^. The spectra of treated specimens were compared with the controls to identify changes in peak positions indicating the presence of new chemical groups. Furthermore, to verify the H-bonding status of the polyester backbone, the 1800–1650 cm^−1^ wavenumber range of all spectra was subjected to curve-fitting analysis (Gaussian area mode) employing PeakFit v.4.12 software (Seasolve, Framingham, MA, USA).

### Statistical analysis

The results of pH and mechanical properties were initially tested for normality (Shapiro–Wilk) and homoscedasticity (Brown–Forsyth) tests. For normally distributed data, comparisons were carried out by one-way ANOVA, whereas for data failed to pass normality tests, the nonparametric one-way ANOVA on Ranks (Kruskal–Wallis) test was used. In all cases, Tukey post hoc multiple comparison tests were used to allocate differences among groups. The level of statistical significance for all tests was set at *a* = 0.05. Statistical analysis was carried out employing SigmaPlot v 14 software (Systat Software Inc, San Jose, CA, USA).

## Results

### pH measurements

The RB and RC cleansers showed a similar pH value (6.31 ± 0.02), whereas the ST cleanser showed a significantly lower pH value (4.83 ± 0.04).

### Mechanical properties (IIT)

**Fig. 1:**
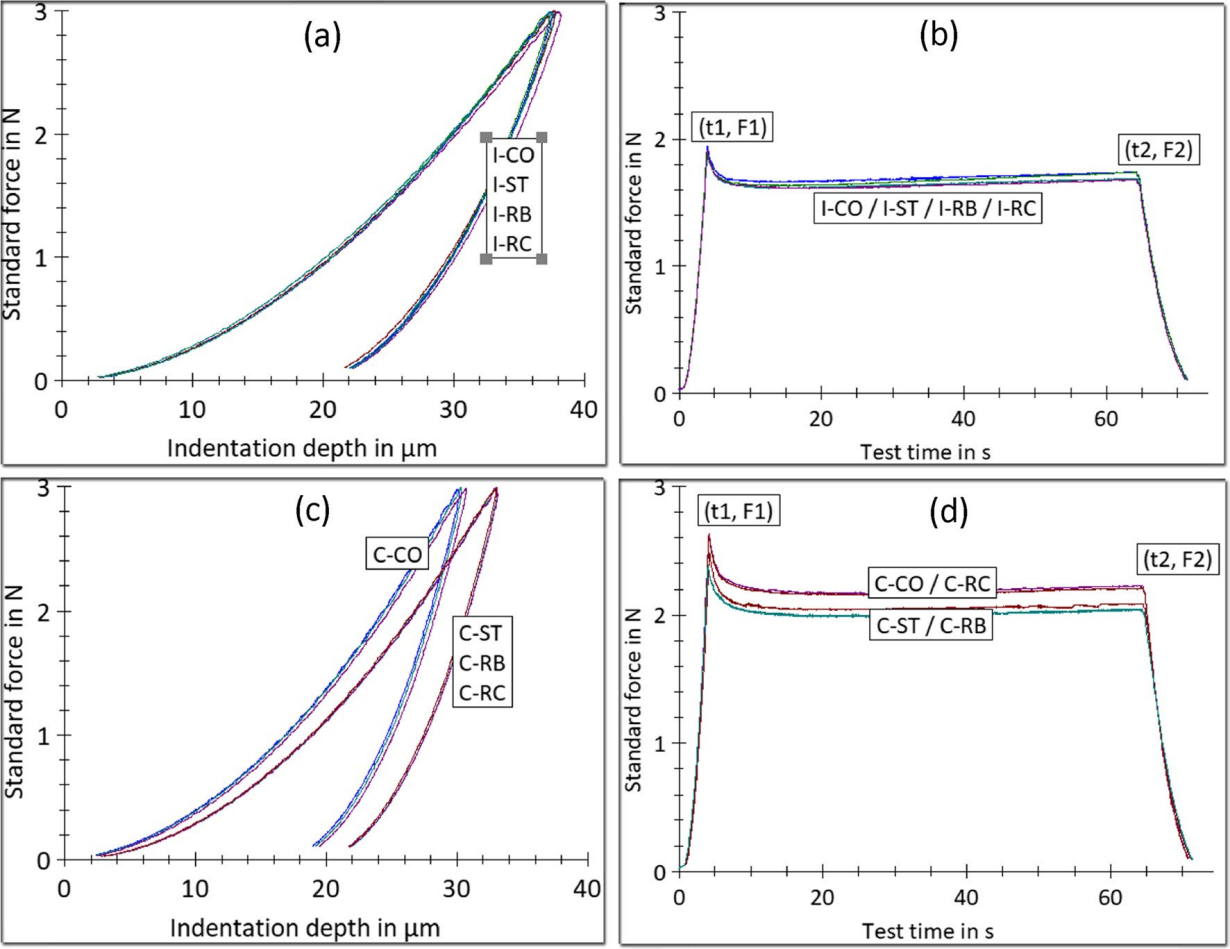
Representative force–indentation depth (**a**, **c**) and force–time curves (**b**, **d**) for Clear Aligner (C) and Invisalign (I) appliances after immersion in Retainer Brite (RB), Retainer Cleaner (RC) and Steraligner (ST) solutions vs the controls (CO)

Figure 1 demonstrates representative force–indentation depth (a, c) and force–time curves (b, d) for the aligners (C, I) per cleaner group (RB, RC, ST) and the control (CO).

For Clear Aligner, a shifting of the peak of the load–indentation graph was found toward higher indentation values after all cleaner treatments in comparison with the control (a), which implies a softening effect. Moreover, two of the cleaner treatments (RB, ST) demonstrated lower force decay overtime from RC and the control (b). The results are summarized in Table 2. All the cleaners comprised a statistically homogeneous group with significantly lower HM, n_IT_ values from the control. Insignificant differences were found between the groups in E_IT_, whereas the R_IT_ measurements revealed significantly reduced values of RB, ST groups from RC and the control (CO).

**Table 2:** The results of the IIT-derived mechanical properties for Clear Aligner (C)

Group	HM (N/mm^2^)	E_IT_ (MPa)	n_IT_ (%)	R_IT_ (%)
C–CO	112 (6)^a^	2699 [2414 2991]	40.6 (0.7)^a^	8.4 [7.9 12.8]^a^
C-RB	106 (3)^b^	2469 [2409 3034]	39.0 (0.6)^b^	15.1 [14.1 15.6]^b^
C-RC	108 (1)^b^	2529 [2352 3041]	39.1 (0.5)^b^	9.0 [8.4 9.2]^a^
C-ST	107 (3)^b^	2466 [2376 2643]	38.6 (0.6)^b^	12.1 [8.7 13.3]^b^

Mean values and standard deviations (in parentheses) or median and 25% and 75% percentiles (in brackets). Same superscript letters show groups without statistical differences per property (*p* > 0.05)

For Invisalign, the loading and unloading curves were identical (a, b) indicating insignificant differences between the cleaner groups tested and the control, as is verified from the numerical data given in Table 3.

**Table 3:** The results of the IIT-derived mechanical properties for Invisalign (I)

Group	HM (N/mm^2^)	E_IT_ (MPa)	n_IT_ (%)	R_IT_ (%)
I-CO	80 (4)	1615 (148)	44.7 [44.2 45.9]	9.3 [6.5 11.4]
I-RB	80 (5)	1605 (141)	43.6 [42.4 44.6]	9.2 [6.9 12.3]
I-RC	79 (4)	1558 (197)	46.0 [45.0 46.6]	8.5 [7.6 9.2]
I-ST	83 (5)	1709 (148)	45.6 [45.2 46.4]	9.6 [5.4 13.8]

Mean values and standard deviations (in parentheses) or median and 25 and 75% percentiles (in brackets). No statistically significant differences were found between the immersion groups and the control for the properties tested (*p* > 0.05)

### Surface chemical composition (ATR-FTIR)

**Fig. 2:**
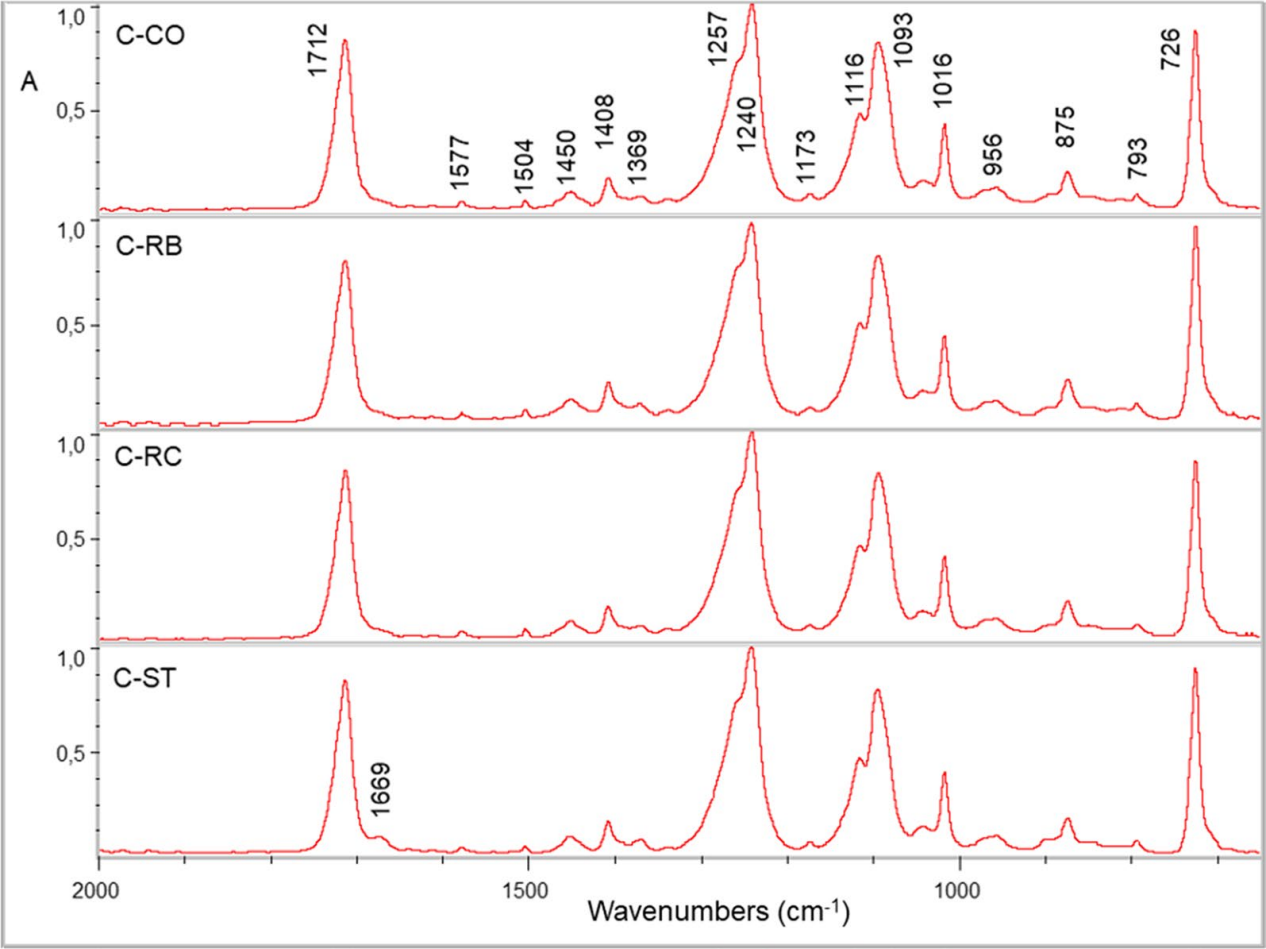
ATR-FTIR spectra of Clear Aligner (C) before (CO) and after treatments with Retainer Brite (RB), Retainer Cleaner (RC) and Steraligner (ST) cleaners. An additional peak appeared at 1669 cm^−1^ after ST cleaner (expanded 2000–650 cm^−1^ range, absorbance scale)

**Fig. 3:**
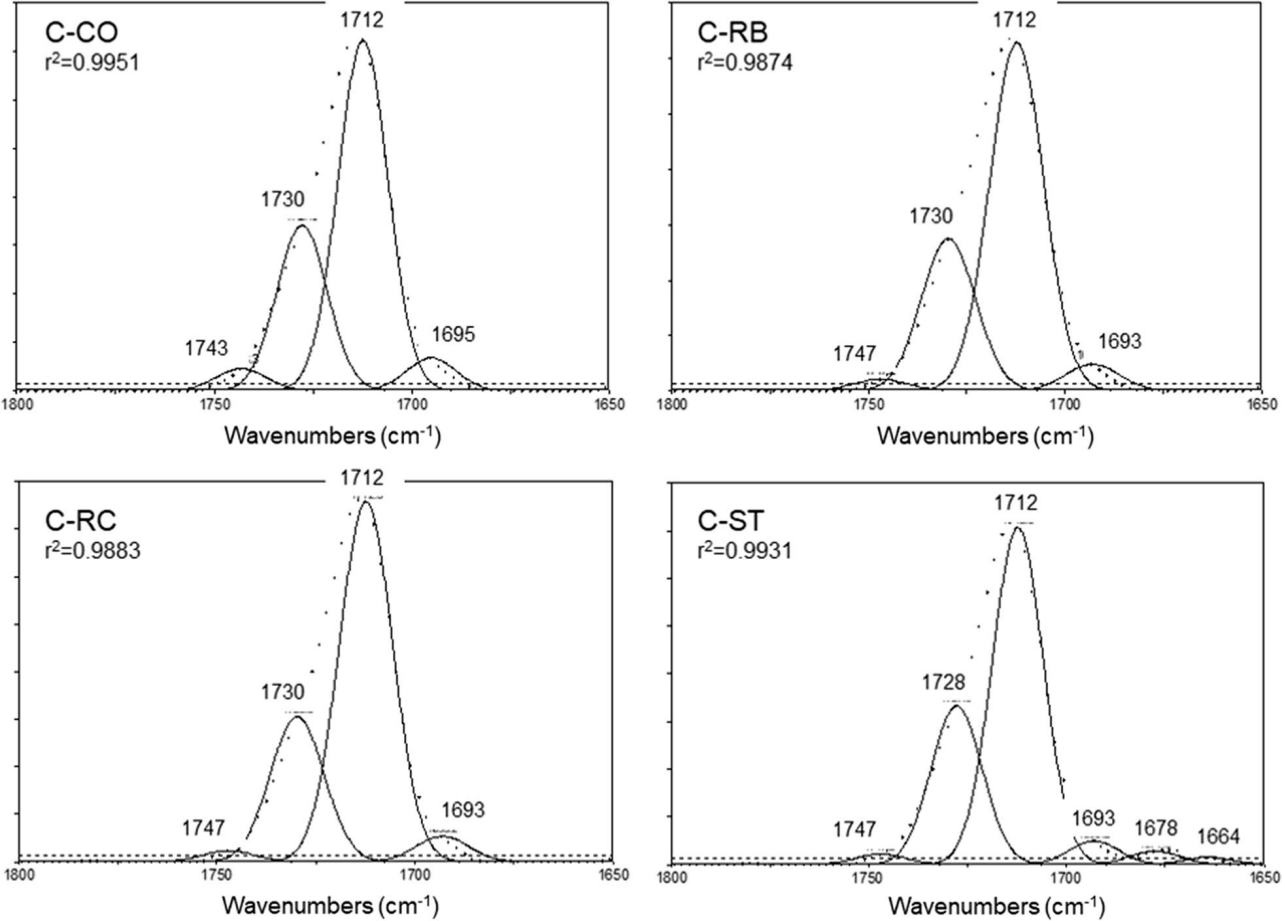
Gaussian curve-fitting of the ester peak of Clear Aligner before (CO) and after treatments with Retainer Brite (RB), Retainer Cleaner (RC) and Steraligner (ST) cleaners. The additional peak after ST cleaner at 1669 cm^−1^ of Fig. 2 is analyzed in two peaks at 1678 cm^−1^ and 1664 indicating formation of acid derivatives (1800–1650 cm^−1^ range, absorbance scale, dotted lines: original spectra, *r*^2^: coefficient of determination for the goodness of curve-fit)

**Fig. 4:**
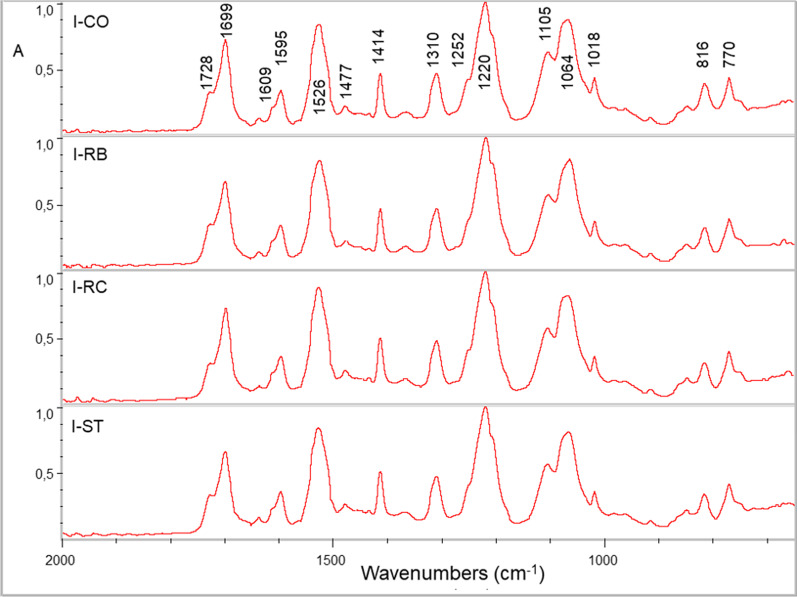
ATR-FTIR spectra of Invisalign (I) before (CO) and after treatments with Retainer Brite (RB), Retainer Cleaner (RC) and Steraligner (ST) cleaners. Spectra are identical (expanded 2000–650 cm^−1^ range, absorbance scale)

Representative ATR-FTIR spectra of the aligners before and after cleaning treatments are illustrated in Figs. 2, 3 and 4.

For Clear Aligner (Fig. 2), the peak assignments are as follows (cm^−1^): 2926 and 2854 (C–H stretching) [not shown in the expanded spectra of the figure]; 1712 (C=O stretching); 1577, 1604 (aromatic C–C stretching); 1450, 1408, 1369 (C–H bending); 1257, 1240 (C=O stretching), 1173 (C–H bending); 1113, 1093 (C–O– stretching); 1016 (C–C ring bending), 956 (C–H stretching of the cyclohexylene ring); 875, 723 (aromatic C–H bending) [17–19]. The cleaning procedures showed similar spectra, except for ST, which demonstrated a small peak at 1670 cm^−1^ assigned to acid groups [20]. The curve-fit analysis of the ester peak of Clear Aligner (Fig. 3 and Table 4) showed two major peaks at 1727 cm^−1^ (free C=O groups) and 1712 cm^−1^ (H–bonded C=O groups) comprising 90–93% of the total C=O peak area (tA_C=O_) at a ratio of 0.4–0.5 (free to H–bonded, based on mean values) for RB, RC and CO, ST, respectively. All specimens showed minor peaks at 1740 cm^−1^ (2–4% of tA_C=O_) and 1693 cm^−1^ (5–6% of tA_C=O_) possibly assigned to oxidation byproducts.

**Table 4:** The results of the curve-fitting analysis of the ester peak for Clear Aligner (C)

Group	Peak area (%)					
	1743 cm^−1^	1727 cm^−1^	1712 cm^−1^	1693 cm^−1^	1677 cm^−1^	1664 cm^−1^
C–CO	3.9	29.1	61.3	5.7	–	–
C-RB	2.1	28.5	64.6	4.8	–	–
C-RC	2.1	26.9	66.2	4.8	–	–
C-ST	1.9	27.7	60.4	4.1	3.4	2.5

1727 cm^−1^: Free C=O groups; 1712 cm^−1^: H–bonded C=O groups; 1743, 1693 cm^−1^: Oxidation byproducts; 1677, 1664 cm^−1^: Acid impurities

The control group demonstrated approximately twice the area of the 1740 cm^−1^ peak in comparison with the treated groups (4 vs 2 for ST and 2.1 for RB, RC), whereas the differences in the 1690 cm^−1^ peak area were smaller (5.7 vs 4.1 for ST and 4.8 for RB, RC). The ST group demonstrated additionally two low wavenumber peaks (1677 and 1644 cm^−1^, 5.9% in sum of tA_C=O_) attributed to acid formation [20].

For Invisalign (Fig. 4), the peak assignments are as follows (cm^−1^): 3330–3270 (N–H stretching); 2927–2919 and 2850 (C–H stretching) [not shown in the expanded spectra of the figure]; 1726–1699 (C=O stretching); 1609 and 1595 (aromatic C–C stretching); 1526 (C–N and N–H bending); 1477, 1412, 1365 (C–H bending); 1310 (C=O vibrations), 1252 (C–N and C–O stretching); 1220, 1105, 1064 and 1017 (C–O–C stretching) 816 and 770 (aromatic C–H bending) [17, 21]. No differences were found after the cleaning treatments and the controls.

**Fig. 5:**
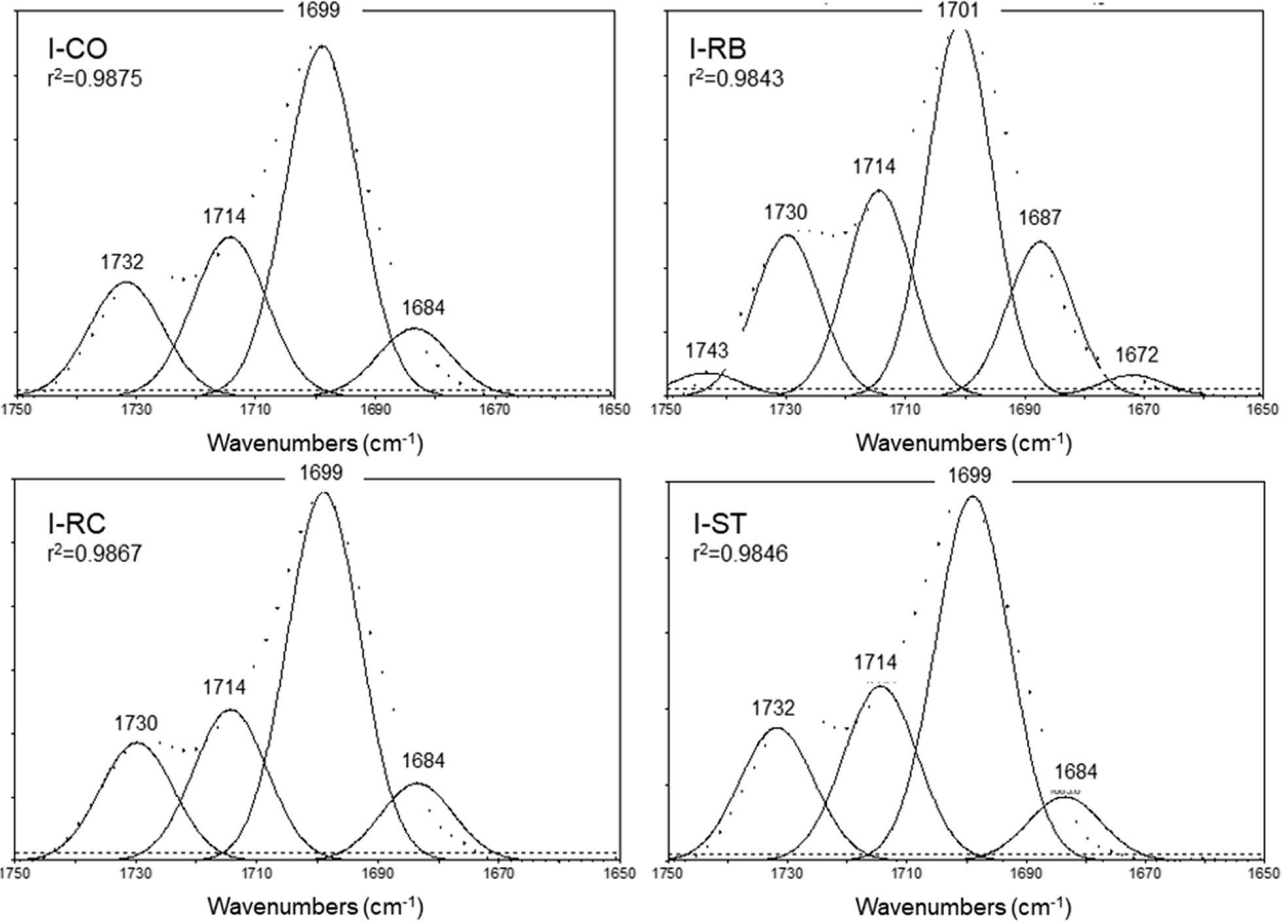
Gaussian curve-fitting of the ester peak of Invisalign (I) before (CO) and after treatments with Retainer Brite (RB), Retainer Cleaner (RC) and Steraligner (ST) cleaners. After RB treatment, two additional peaks appeared (1743 cm^−1^, 1672 cm^−1^) and the intensity of the peak at 1687 cm^−1^ was increased indicating changes in the H–bonding status of the ester groups (1750–1650 cm^−1^ range, absorbance scale, dotted lines: original spectra, *r*^2^: coefficient of determination for the goodness of curve-fit)

The curve-fit analysis of the ester peak of Invisalign (Fig. 5 and Table 5) resolved four peak components assigned to polyurethane (hard polymer segment) or polycarbonate (soft polymer segment) of poly(ester–urethane) polymers at 1732 cm^−1^ (free C=O groups of urethane and carbonate components), 1714 cm^−1^ (H–bonded C=O groups of carbonate component), 1699 cm^−1^ (H–bonded C=O groups of amorphous urethane component) and 1683 cm^−1^ (H–bonded C=O groups of low-ordered urethane component) [22]. The free C=O accounted for 16.7–18.2% of the tA_C=O_ (mean values) and were not affected by the treatments. The same applied for the H–bonded C=O groups of the carbonate segment (21.7–23.8%). However, for the amorphous urethane H–bonded C = O groups, a reduction in the peak area was found after RB treatment (39.8%) in comparison with the control (50.5%) and the other treatments (50.3% for RC and 44.3% for ST). This difference was in favor of the low-ordered urethane H–bonded C=O groups, which increased after RB treatment (16.3%) in comparison with the control (9.8%), RC (11%) and ST (8.6%).

**Table 5:** The results of the curve-fitting analysis of the ester peak for Invisalign (I)

Group	Peak area (%)					
	1743 cm^−1^	1732 cm^−1^	1714 cm^−1^	1699 cm^−1^	1684 cm^−1^	1672 cm^−1^
I-CO	–	16.7	23	50.5	9.8	–
I-RB	2.5	17.2	21.9	39.8	16.3	2.3
I-RC	–	17	21.7	50.3	11	–
I-ST	–	18.2	23.8	44.3	8.6	–

1732 cm^−1^: Free C=O groups of urethane and carbonate; 1714 cm^−1^: H–bonded C=O groups of urethane and carbonate; 1699 cm^−1^: H–bonded C=O groups of amorphous urethane segments; 1684 cm^−1^: H–bonded groups of low-ordered urethane segments; 1743, 1672 cm^−1^: Acid impurities

## Discussion

The orthodontic force delivered by aligners depends on material thickness, hardness, elastic modulus and amount of activation [1]. To predictably move teeth, it is important that the mechanical properties of the aligners to be stable during the in-service period [2]. However, during usage the aligners are not only exposed to the oral environment, but should be treated with disinfecting and cleaning solution for hygienic purposes [3–7] The results of the present study showed that some cleaners may affect the mechanical properties or/and the surface chemistry of aligner materials fabricated by polyethylene terephthalate glycol (PET-G) or poly(ester–urethane). Consequently, the null hypothesis should be partially rejected.

The cleaners tested have been specifically designed for orthodontic aligners, although the quantitative composition of some (RB, RC) resembles that of conventional denture-base cleaners [23]. RB and RC cleaners are mainly composed of sodium perborate or sodium percarbonate, which in water solutions decompose to borates and hydrogen peroxide or hydrogen peroxide with sodium and carbonate ions. The hydrogen peroxide further decomposes to active oxygen and water, whereas the carbonates to carbon dioxide and water [24]. The cleansers contain surfactants, flavoring agents and pigments. It has been documented that the cleaners of this category (commonly referred to as alkaline peroxides) reduce the hardness and flexural strength and increase the roughness of polymethyl methacrylate denture-base materials through hydrolytic oxidation and network plasticization (extraction of residual methyl methacrylate monomer, cross-linkers, oxidation byproducts, etc.) [25, 26]. For ST, the composition given does not define any source of active oxygen as in the other two cleaners, the only difference being the lower pH. All the cleaners contain EDTA chelators. EDTA is known to inhibit biofilm formation, especially the tetrasodium salt [27], whereas the disodium demonstrates increased solubility in water and a faster chelation effect [28]. Also, pyrophosphates and polyphosphates are used as inhibitors of Ca and Mg precipitation on the appliances [29, 30].

In the present study, none of the mechanical properties of the Invisalign aligners showed significant difference after immersion in any of the three cleaning solutions in comparison with the control group. This implies that the poly(ester–urethane) structure of Invisalign was stable to the degradative effects of the cleaners tested. However, for Clear Aligner, a significant reduction in Martens Hardness and elastic index was manifested, which indicates that these aligners became softer and more brittle, irrespectively of the active ingredients and the pH of the cleaners used. A possible explanation is an increased hydrolytic instability of the esterified hydrophilic polyglycol segments of the amorphous PET-G. An interesting finding of the study was the different effects of RB and RC cleaners in R_IT_, which is associated with the behavior of the aligner materials under creep. RC with the same pH with RB was less aggressive to Clear Aligner, matching the effect of the control group, while RB demonstrated significantly higher R_IT_ values from RC, being similar with the acidic ST. This may suggest that several compositional factors, other than the pH, may induce the hydrolytic degradation. In industrial applications, sodium percarbonate is considered more reactive than sodium perborate, with the latter requiring additional alkalinity (usually mediated by solutions of 1% NaOH) for an effective bleaching effect [31]. It is not known if a similar mechanism is implemented in RB, which would explain the difference. Furthermore, a parameter which may affect the mechanical properties of the aligners is the undefined role of residual stresses developed during the manufacturing process [32]. Successive immersion may provide an extent of relaxation with a subsequent effect on the mechanical properties measured [32]. However, the extent and orientation of residual stresses of orthodontic retainers still remain unknown. This may be an interesting topic for further research.

To characterize the chemical changes induced on the aligner surfaces, an ATR-FTIR analysis was used. Although the sampling depth of the method is limited to the uppermost 2 μm zone (vs bulk characterization of ground/polished specimens by IIT), it may provide important information on the degradation mechanisms involved. Comparison of the spectra at the fingerprint range (2000–650 cm^−1^) showed a difference only in Clear Aligner treated with ST, where a peak appeared at 1699 cm^−1^ attributed to acid production via oxidation of the PET structure [20]. A more detailed analysis by curve-fitting of the ester peak components demonstrated the presence of free and H–bonded C=O groups at a ratio of 0.4 for RB, RC and 0.5 for CO and ST. The small reduction observed after treatment with the alkaline peroxides (RB, RC) may suggest degradation of a small fraction of free-ester groups. The highest and lowest wavenumber weak peaks (1743, 1693 cm^−1^) found in all groups indicate that oxidized impurities existed in the control and where reduced after treatments, mainly at the highest wavenumber. The two additional peaks resolved at 1677 and 1644 cm^−1^ suggest that the acid produced may appear in more than one forms (terephthalic, glycolic, etc.). Considering the depth of the ATR and IIT methods, it may be concluded that the chemical changes may exceed up to the depth of the IIT method, affecting the mechanical properties accordingly. Curve-fitting of the ester peak components revealed some interesting information for Invisalign, the mechanical properties of which were not affected by any of the cleaners, as documented by the fingerprint range spectra. After RB treatment, weak highest (1740 cm^−1^) and lowest (1672 cm^−1^) wavenumber peaks appeared indicating oxidative effects, while the peak at 1687 cm^−1^ assigned to the low-ordered crystallinity of the urethane segment was increased at the expense of the corresponding amorphous (1699 cm^−1^). This may indicate an onset of the development of a more brittle structure, possibly associated with aging. The fact that this phenomenon was observed only after treatment with one alkaline peroxide (RB), suggests that the poly(ester–urethane) structure is more sensitive to this type of cleaners and that RB is a stronger alkaline peroxide than RC. Although the chemical changes of Invisalign were not associated with the mechanical response, they clearly demonstrate the capacity of ATR-FTIR spectrometry in identifying early degradative changes in the surface chemistry of the aligners.

The results of the present study should be carefully interpreted since the aligners were not subjected to intraoral conditions, to reliably assess the extent of the cleaner-induced degradation in the performance of intraorally exposed analogues. Such changes, though, documented in simple immersion tests may contribute to the earlier deterioration of the aligner properties, possibly affecting the in-service life of the devices. Further studies, considering in vivo functional loading as a testing factor may enlighten the role of the cleansers to the properties of the aligners and facilitate defining the onset of the mechanical deterioration of these thermoformed materials.

## Conclusion

The mechanical properties of the Invisalign aligners devices did not change after immersion in the cleaning solutions (two alkaline peroxides and one acidic), whereas Clear Aligner devices showed evidence of softening and brittleness (all solutions) and increased relaxation in two solutions (alkaline peroxide and acidic). However, these changes may be implicated with residual stresses.

The surface chemical analysis revealed acid formation in Clear Aligner after the acidic treatment, whereas the H–boned status analysis of Clear Aligner ester groups manifested a small reduction of the free-ester groups after alkaline peroxide treatments. Changes in Invisalign surface chemistry were registered only for one alkaline cleaner by the H–bonding status analysis of the ester groups, suggesting early signs of degradation.

Based on these findings, the cleaners tested should be used with caution in PET-G aligners, while some alkaline peroxide solutions should be avoided for cleaning poly(ester–urethane) aligners.

## Data Availability

The datasets used and/or analyzed during the current study are available from the corresponding author on reasonable request.
